# Effects of ageing on pro-arrhythmic ventricular phenotypes in incrementally paced murine *Pgc-1β*^*−/−*^ hearts

**DOI:** 10.1007/s00424-017-2054-3

**Published:** 2017-08-18

**Authors:** Shiraz Ahmad, Haseeb Valli, Charlotte E. Edling, Andrew A. Grace, Kamalan Jeevaratnam, Christopher L-H Huang

**Affiliations:** 1 Physiological Laboratory, University of Cambridge, Downing Street, Cambridge, CB2 3EG UK; 2 Faculty of Health and Medical Sciences, University of Surrey, Guildford, Surrey GU2 7AL UK; 3 Department of Biochemistry, University of Cambridge, Tennis Court Road, Cambridge, CB2 1QW UK; 4 PU-RCSI School of Medicine, Perdana University, 43400 Serdang, Selangor Darul Ehsan Malaysia

**Keywords:** Peroxisome proliferator-activated receptor-γ coactivator-1 (PGC-1), Ventricles, Action potential, Wavelength, Cardiac conduction, Cardiac arrhythmias

## Abstract

A range of chronic clinical conditions accompany cardiomyocyte energetic dysfunction and constitute independent risk factors for cardiac arrhythmia. We investigated pro-arrhythmic and arrhythmic phenotypes in energetically deficient C57BL mice with genetic ablation of the mitochondrial promoter peroxisome proliferator-activated receptor-γ coactivator-1β (*Pgc-1β*), a known model of ventricular arrhythmia. Pro-arrhythmic and cellular action potential (AP) characteristics were compared in intact Langendorff-perfused hearts from young (12–16 week) and aged (> 52 week), wild-type (WT) and *Pgc-1β* ^*−/−*^ mice. Simultaneous electrocardiographic and intracellular microelectrode recordings were made through successive trains of 100 regular stimuli at progressively incremented heart rates. Aged *Pgc-1β* ^*−/−*^ hearts displayed an increased incidence of arrhythmia compared to other groups. Young and aged *Pgc-1β* ^*−/−*^ hearts showed higher incidences of alternans in both AP activation (maximum AP upshoot velocity (d*V*/d*t*)_max_ and latency), recovery (action potential duration (APD_90_) and resting membrane potential (RMP) characteristics compared to WT hearts. This was particularly apparent at lower pacing frequencies. These findings accompanied reduced (d*V*/d*t*)_max_ and increased AP latency values in the *Pgc-1β* ^*−/−*^ hearts. APs observed prior to termination of the protocol showed lower (d*V*/d*t*)_max_ and longer AP latencies, but indistinguishable APD_90_ and RMPs in arrhythmic compared to those in non-arrhythmic hearts. APD restitution analysis showed that *Pgc-1β* ^*−/−*^ and WT hearts showed similar limiting gradients. However, *Pgc-1β* ^*−/−*^ hearts had shortened plateau AP wavelengths, particularly in aged *Pgc-1β* ^*−/−*^ hearts. *Pgc-1β* ^*−/−*^ hearts therefore show pro-arrhythmic instabilities attributable to altered AP conduction and activation rather than recovery characteristics.

## Introduction

Following the successful mechanistic and mathematical description of the cardiac action potential and its propagation, cardiac electrophysiology has increasingly focused upon the physiological mechanisms underlying arrhythmia [19]. The risk of cardiac rhythm abnormalities accumulates with age. The prevalence of atrial fibrillation rises from ~ 4% of individuals aged 60–70 to ~ 20% at > 80 years [54], while the incidence of sudden cardiac death attributable to ventricular arrhythmias is eight times higher in 75- than 50-year-old individuals [8]. Metabolic abnormalities associated with chronic, age-dependent, conditions including obesity, insulin resistance, diabetes mellitus and heart failure also accentuate arrhythmic risk, independently of any ischaemic changes arising from associated coronary vascular effects [1, 28, 51]. These situations are accompanied by cardiomyocyte energetic and therefore, mitochondrial dysfunction, itself an independent arrhythmic risk factor [2]. Thus, inherited mitochondrial disorders such as Kearns-Sayre syndrome predispose to fatal ventricular arrhythmias [24]. Fibrotic defects of the cardiac conduction system leading to heart block are similarly age-related and contribute to age-dependent onsets of inherited arrhythmic conditions including the Brugada syndrome [22].

The peroxisome proliferator-activated receptor-γ coactivator-1 (PGC-1) family of transcriptional coactivators offers a strategic target for studying the electrophysiological consequences of energetic deficiency. PGC-1 regulates mitochondrial mass, function and cellular metabolism [13], upregulating nuclear and mitochondrial gene expression involved in fatty acid β-oxidation, the tricarboxylic acid cycle and the electron transport chain [3]. PGC-1 protein expression and, correspondingly, mitochondrial activity vary with upstream cellular energy demand [44]. Metabolic conditions such as obesity, insulin resistance and type II diabetes as well as advanced age are associated with reduced PGC-1 protein expression and mitochondrial dysfunction [9, 30, 36].

Much of the biochemical insight into the consequences of cardiac energetic deficiency [21, 38] derives from studies in hearts with dysfunctional PGC-1 networks [4, 20]. *Pgc-1α* ^*−/−*^ murine hearts show normal baseline contractility, developing heart failure only with increased afterload [3]. *Pgc-1β* ^*−/−*^ hearts similarly show normal baseline cardiac function, but display compromised heart rate responses with adrenergic stimulation [29]. Langendorff-perfused *Pgc-1β* ^*−/−*^ hearts demonstrated preliminary evidence for increased arrhythmogenicity. Their isolated cardiomyocytes showed diastolic Ca^2+^ transients, afterdepolarisation events and altered ion channel expression patterns [17], abnormalities also known to occur with ageing [18].

The combination of normal contractile function with pro-arrhythmic electrophysiological changes suggests that *Pgc-1β* ^*−/−*^ hearts are suitable models to explore the pro-arrhythmic effects of mitochondrial impairment. Isolated, perfused, murine hearts have proved to be useful in the study of arrhythmic phenotypes and their underlying mechanisms. They have been particularly important in studies of specific genetic modifications directed at well-defined, inherited, monogenic ion channelopathies [19, 22, 25]. The present study extends these analyses to *Pgc-1β* ^*−/−*^ hearts through the analysis of the electrophysiological consequences of energetic dysfunction. Chronic mitochondrial lesions likely exert cumulative and time-varying phenotypic effects with advancing age, and so the experiments studied both young and aged, wild-type (WT) and genetically modified animals.

## Methods

### Animals

This research has been regulated under the Animals (Scientific Procedures) Act 1986 Amendment Regulations 2012 following ethical review by the University of Cambridge Animal Welfare and Ethical Review Body (AWERB). The experiments also conformed to the Guide for the Care and Use of Laboratory Animals, U.S. National Institutes of Health (NIH Publication No. 85-23, revised 1996). Mice were housed in an animal facility at 21 °C with 12-h light/dark cycles. Animals were fed sterile chow (RM3 Maintenance Diet; SDS, Witham, Essex, UK) and had free access to water, bedding and environmental stimuli. Mice were sacrificed by cervical dislocation. No recovery, anaesthetic or surgical procedures were required. Wild-type (WT) C57/B6 and *Pgc-1β* ^*−/−*^ adult mice were bred for the experimental protocols. *Pgc-1β* ^*−/−*^ mice were generated as described previously [17, 29]. Briefly, a triple LoxP targeting vector was used, containing a floxed neomycin phosphotransferase selectable marker cassette inserted into intron 3 and a single LoxP site inserted into intron 5. This resulted in the deletion of exons 4 and 5. The presence of LoxP sites was confirmed in embryonic stem cells using southern blot analysis. Heterozygous triple LoxP mice crossed with ROSA26-Cre mice generated heterozygous *Pgc-1β* ^*−/−*^ mice, which were further bred to generate homozygous *Pgc-1β* ^*−/−*^ mice. Mice were bred on a C57/B6 background to avoid possible strain-related confounds. Experiments were performed in four experimental groups: young WT (*n* = 23), young *Pgc-1β* ^*−/−*^ (*n* = 21), aged WT (*n* = 19) and aged *Pgc-1β* ^*−/−*^ (*n* = 25). All young mice were aged between 12 and 16 weeks and aged mice greater than 52 weeks.

### Buffering media

All solutions used were based on Krebs–Henseleit (KH) solution (mM: NaCl, 119; NaHCO_3_, 25; KCl, 4.0; KH_2_PO_4_, 1.2; MgCl_2_, 1.0; CaCl_2_, 1.8; glucose, 10; and Na-pyruvate, 2.0; pH adjusted to 7.4 and bubbled with 95% O_2_/5% CO_2_ (British Oxygen Company, Manchester, UK). Chemical agents were purchased from Sigma-Aldrich (Poole, UK). A modified KH solution containing KH mixed with 20 μM blebbistatin (Selleckhem, Suffolk, UK) [10–12, 23] was used for perfusion to immobilise hearts prior to plain Krebs–Henseleit perfusion.

### Whole-heart intracellular microelectrode recordings

Prior to sacrifice, 200 IU of unfractionated heparin (Sigma-Aldrich, Poole, UK) was administered intraperitoneally. Animals were then sacrificed and rapid sternectomy and cardiectomy were performed. All hearts were macroscopically unremarkable with no obvious abnormalities. Hearts were rapidly cannulated and secured as previously described [33, 39, 52] before being mounted on a horizontal Langendorff apparatus that was electrically insulated and incorporated into an intracellular rig within a Faraday cage, incorporating a light microscope (objective ×5, eyepiece ×5; W. Watson and Sons Limited, London, UK), organ bath and custom-built microelectrode amplifier and head stage.

Hearts were mounted in a standard anatomical position to allow impalement of the left ventricular mass and pacing from the right ventricle simultaneously. The positioning of the recording and stimulating electrodes was controlled by two precision micromanipulators (Prior Scientific Instruments, Cambs., UK), calibrated to permit impalements over the same region of the myocardium. Stimulating and recording electrodes were positioned at consistent sites against the lateral surface of the right ventricle and the proximal left ventricle respectively to avoid confounds of regional differences in action potential (AP) characteristics. Accordingly, in any given experiment, alterations in AP latencies, measured as the time elapsed between the pacing stimulus and peak of the AP, reflected alterations in AP conduction velocities. Hearts were perfused with KH solution (at a constant flow rate of 2.1 ml min^−1^) for at least 5 min to reach steady state, before being perfused with KH solution and 20 μM blebbistatin to minimise motion, before resumption of perfusion with plain KH solution. Preparations which did not show a regular intrinsic rhythm with a basic cycle length (BCL) of <200 ms and 1:1 atrioventricular (AV) conduction for > 10-min post-cannulation were not studied further. A conventional sharp 3-M KCl-filled microelectrode (tip resistance 15–25 MΩ, filled with 3 M KCL) was pulled by a custom-built microelectrode puller from a glass pipette (1.2 mm OD, 0.69 mm ID; Harvard Apparatus) and then inserted into a right-angled microelectrode holder (Harvard Apparatus, Kent, UK). This was connected to the headstage of a high-input impedance direct-current microelectrode amplifier system (University of Cambridge, Cambridge, UK). Following band-pass filtering between 0 and 2 kHz and amplification, the signal was passed through an analogue to digital converter at a sampling frequency of 10 kHz (1401; Cambridge Electronic Design, Cambs., UK). Successful impalement was indicated by the abrupt appearance of a resting membrane potential lower than − 70 mV and regular action potentials of stable waveform. All measurements of intracellular voltage were made relative to the bath solution via a reference Ag/AgCl electrode.

### Whole-heart electrocardiographic recordings

Electrocardiographic (ECG) recordings of isolated Langendorff-perfused hearts were obtained simultaneously with intracellular microelectrode measurements to enable correlations between whole-organ rhythms and intracellular voltage changes. Recordings were made using two unipolar ECG electrodes placed at fixed positions around the heart in the organ bath, corresponding to the standard three-lead ECG coordinates. The recorded signals were passed through headstages for pre-amplification, prior to amplification (Neurolog NL104 amplifier) and band-pass filtering (between 5 and 500 Hz; NL 125/6 filter; Digitimer, Herts, UK) before being digitized at a sampling frequency of 10 kHz (1401; Cambridge Electronic Design).

### Pacing protocols

Hearts were stimulated at two times the threshold voltage plus 0.5 mV. Hearts underwent two pacing protocols. A standardised S1S2 protocol consisted of trains of eight stimuli delivered 125 ms apart (S1 stimuli) followed by an extrasystolic (S2) stimulus delivered initially at 90 ms after the previous S1 stimulus. This pattern of stimulation was repeated with the S2-S1 interval decremented by 1 ms for each successive cycle until failure of capture. Incremental pacing protocols began at a 130 ms basic cycle length (BCL) before being decremented by 5 ms for each subsequent cycle of 100 stimulations. This cycle was repeated until the heart showed entry into 2:1 block or arrhythmia.

### Data and statistical analysis

Data was captured with Spike2 software (Cambridge Electronic Design) and then analysed using a custom-written program using the python programming language. Alternans was defined as beat to beat changes in the value of a parameter such that the direction of the change oscillates for at least ten successive action potentials. All statistical analysis was carried out using the R programming language [40] and plots with the grammar of graphics package [49]. All data is expressed as mean ± standard error of mean (SEM) and a *p* value of less than 0.05 taken to be significant. The different experimental groups were compared with a two-factor analysis of variance (ANOVA). *F* values that were significant for interactive effects prompted post hoc testing with Tukey honestly significant difference testing. If single comparisons were made, a two-tailed Student’s *t* test was used to compare significance. Categorical variables were compared using Fisher’s exact test. Kaplan-Meier estimates were compared with the log-rank test.

## Results

We performed simultaneous electrocardiographic (ECG) whole-heart recordings and action potential (AP) measurements from single cardiomyocytes in intact ex vivo Langendorff-perfused hearts. Each procedure began with a programmed electrical stimulation protocol interposing extrasystolic (S2) stimuli at successively decremented intervals following trains of eight regular (S1) stimuli applied at a 125 ms basic cycle length (BCL). Overall ventricular electrical activity through the S1S2 protocol was monitored by the ECG recordings. Analysis of these initial recordings yielded ventricular effective refractory periods (VERPs) that indicated the extent to which BCLs could be decreased in the succeeding experiments. A comparison of VERPs in young and aged, WT (57.36 ± 2.06 and 56.3 ± 1.96 ms, respectively) and *Pgc-1β* ^*−/−*^ (53.99 ± 2.2 and 47.64 ± 1.84 ms, respectively) hearts demonstrated that *Pgc-1β* ^*−/−*^ hearts showed shorter VERPs than WT hearts (56.88 ± 1.42 vs 50.54 ± 1.48 ms; *n* = 42 vs 46; *p* < 0.01), without effects of either age or interactions between age and genotype.

**Fig. 1:**
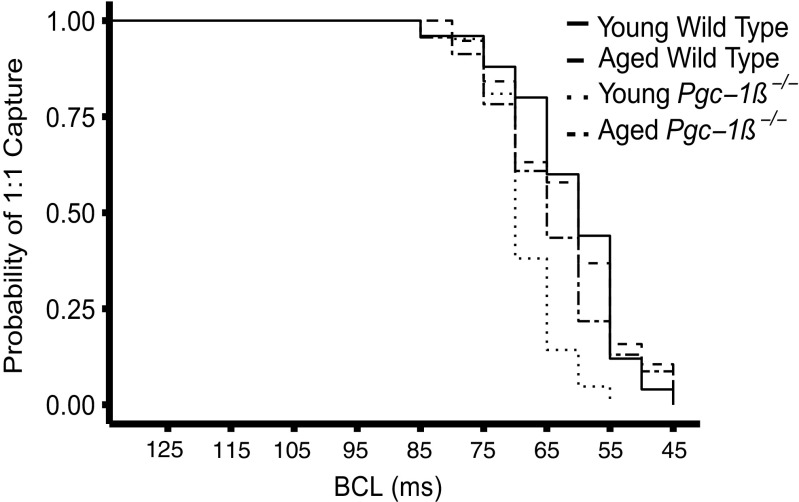
Kaplan-Meier plot of the proportion of young and aged WT and *Pgc-1β* ^*−/−*^ hearts showing regular activity with increasing pacing frequency with the progressive fall off at higher pacing frequencies resulting in hearts losing capture and entering either 2:1 conduction block or arrhythmia

Intracellular microelectrode recordings of cardiomyocyte APs within left ventricular epicardia were then obtained in parallel with ECG recordings of isolated Langendorff hearts during an incremental pacing protocol. Trains of 100 regular stimuli were imposed at BCLs progressively shortened by 5 ms with each successive stimulus train. These examined for the presence of arrhythmic activity and alternans in AP parameters related to their activation or recovery. Figure 1 tracks the progress of the experiments through the incremental pacing protocol in all the four groups, using Kaplan-Meier plots of the probability of hearts showing regular 1:1 evoked activity with decreasing BCL. Quantitative analysis was performed for BCLs up to 55 ms, after which insufficient hearts showed regular 1:1 capture for analysis. The BCLs at which hearts failed to follow the repetitive pacing and entered 2:1 block reflect their VERP at that particular pacing rate. A log-rank test demonstrated that the Kaplan-Meier curves from the different experimental groups (Fig. 1) showed significant differences from each other (*p* < 0.01). VERPs at the termination of the protocol (young WT, 64.35 ± 2.16 ms; aged WT, 61.84 ± 2.33 ms; young *Pgc-1β* ^*−/−*^, 68.33 ± 1.26 ms; aged *Pgc-1β* ^*−/−*^ hearts, 61.0 ± 1.83 ms) were obtained from the BCLs achieved just prior to the onset of 2:1 atrioventricular conduction block.

### Age-dependent arrhythmic phenotypes in *Pgc-1β*^*−/−*^ hearts

**Fig. 2:**
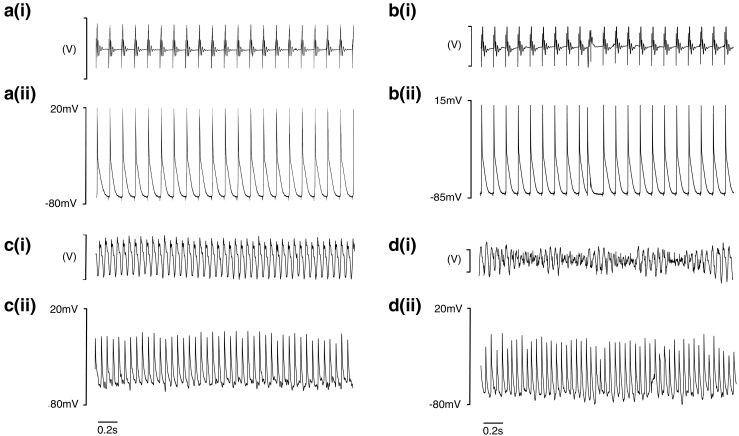
**a**–**d** Parallel ECG (i) and intracellular AP recordings (ii) of regular activity at a 130 ms BCL in ventricles from heart from young WT mouse (**a**), and ectopic beat (**b**), monomorphic VT (**c**) and torsades de pointes (TdP) (**d**) VT in aged *Pgc-1β* ^*−/−*^ hearts

Figure 2 exemplifies intracellular electrophysiological traces of normal, arrhythmic and pro-arrhythmic activities from all the four experimental groups obtained in the course of the incremental pacing protocol. Figure 2 compares parallel ECG (i) and intracellular AP recordings (ii) of regular ventricular activity at a BCL of 130 ms from a young WT mouse (a). Similar simultaneous ECG (i) and intracellular AP recordings (ii) recordings made it possible to detect ectopic beats (b), monomorphic ventricular tachycardia (VT) (c) and torsades de pointes (TdP) (d) in aged *Pgc-1β* ^*−/−*^ hearts. Under these experimental conditions, there were no observations of spontaneous early or delayed afterdepolarisations. The latter have been reported in other pro-arrhythmic murine systems where they have been implicated in arrhythmic triggering [5, 6, 14].

Table 1 quantifies the occurrence of arrhythmic phenomena in cardiomyocytes from which the intracellular records were obtained. Aged *Pgc-1β* ^*−/−*^ hearts showed the highest arrhythmic incidences compared to the remaining groups (*p* < 0.05). The latter groups showed similar, reduced, arrhythmic frequencies. Aged *Pgc-1β* ^*−/−*^ hearts were also the only experimental group that showed ectopic activity, monomorphic VT and polymorphic VT (Fig. 2b–d). The incidence of arrhythmias in hearts from young *Pgc-1β* ^*−/−*^ hearts was not distinguishable from their WT counterparts.

**Table 1:** Frequency of arrhythmia in the various experimental groups

	Arrhythmic	Non-arrhythmic	Total (*n*)	Percentage arrhythmic
Young WT	4	19	23	17.4
Aged WT	3	16	19	15.8
Young *Pgc-1β* ^*−/−*^	4	17	21	19.0
Aged *Pgc-1β* ^*−/−*^	12*	13	25	48*

*p < 0.05

**Fig. 3:**
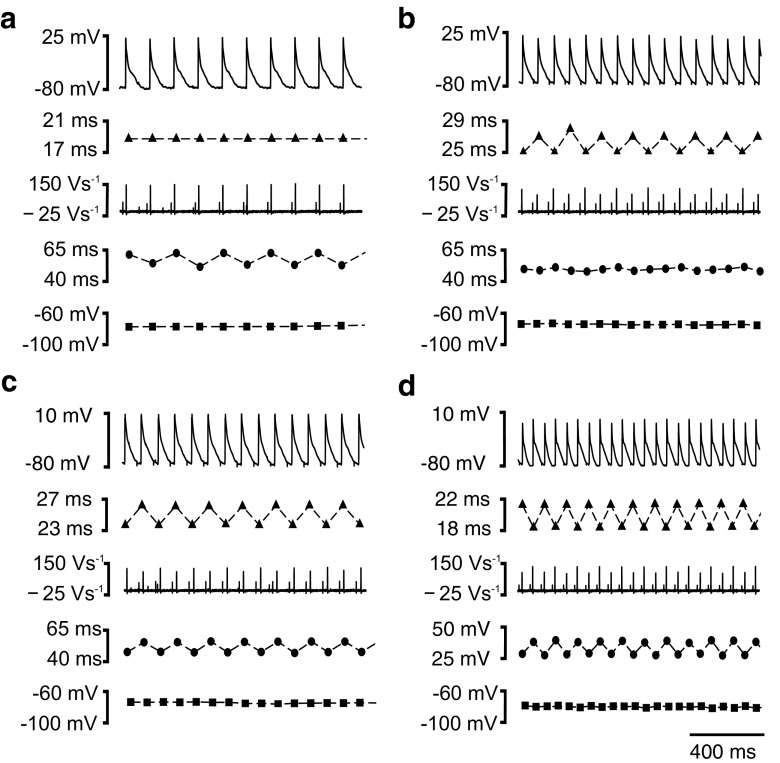
Examples of alternans phenomena comparing records of AP waveform, AP latency, (d*V*/d*t*), APD_90_ and RMP respectively in each panel. In **a**, alternans exclusively involves the recovery variable APD_90_ while in **b**, it involves the activation variables of AP latency and (d*V*/d*t*) alone. In **c** and **d**, there is simultaneous alternans involving both activation and recovery variables. These include situations in which the alternating action potentials with higher/lower latencies and therefore lower/higher (d*V*/d*t*)_max_ show either higher/lower (**c**) or lower/higher (**d**) APD_90_, respectively

Figure 3 exemplifies the different forms of alternans in AP characteristics observed in a typical aged *Pgc-1β* ^*−/−*^ heart with each panel displaying (from top to bottom) AP waveforms, AP latencies, the first derivative, d*V*/d*t*, of the AP waveform, APD_90_ and RMP. This makes it possible to directly visualize alternans in one or more electrophysiological parameters. The panels demonstrate the possible combinations of alternans involving activation or recovery parameters. Thus, the alternans in (a) involves the recovery variable of APD_90_ alone while (b) demonstrates alternans in the activation variables of AP latency and (d*V*/d*t*)_max_ alone. In contrast, (c) and (d) exemplify simultaneous alternans involving both activation and recovery variables. They include the extreme situations resulting in alternate APs with higher/lower latencies and therefore lower/higher (d*V*/d*t*)_max_ associated with higher/lower (c) or lower/higher APD_90_ (d). The second case (d) would correspond to the most pro-arrhythmic situation. The bottom control trace shows the (relatively constant) resting potentials through the alternans phenomena.

### Increased frequencies of alternans in *Pgc-1β*^−/−^ hearts

**Fig. 4:**
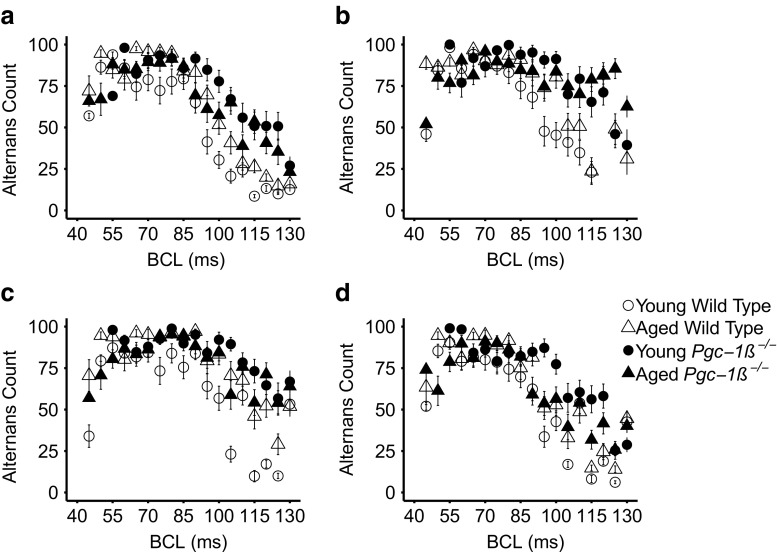
Incidence of alternans out of 100 beats at each BCL in the activation variables of maximum AP upstroke rate (d*V*/d*t*)_max_ (**a**), AP latency (**b**), and the recovery variables of AP duration to 90% recovery (APD_90_) (**c**) and resting membrane potential (RMP) (**d**) in young and aged, WT and *Pgc-1β* ^*−/−*^ hearts through the incremental pacing protocol

The presence of alternans in one or more AP characteristics with variations in BCL is an established marker for the instability presaging development of arrhythmia (see Introduction). We first compared the number of beats showing alternans in maximum AP upstroke rate (d*V*/d*t*)_max_ (Fig. 4a), AP latency (Fig. 4b), AP duration to 90% recovery (APD_90_) (Fig. 4c) and resting membrane potential (RMP) (Fig. 4d) in young and aged, WT and *Pgc-1β* ^*−/−*^ hearts through the incremental pacing protocol described above. Overall incidences of alternans were obtained for each heart by summing the number of beats of alternans at each BCL examined. The results were then compared between the experimental groups. *Pgc-1β* ^*−/−*^ hearts showed greater overall incidences of alternans compared to WT hearts in all the parameters (d*V*/d*t*)_max_, AP latency, APD_90_ and RMP. Thus, both young and aged *Pgc-1β* ^*−/−*^ hearts displayed an increased tendency to display alternans (Table 2).

**Table 2:** Results of ANOVA comparisons of alternans features in *Pgc-1β* ^*−/−*^ and WT hearts. Values represent number of beats out of 1600 beats

	AP parameter	*Pgc-1β* ^*−/−*^	WT	*p* value
Overall incidences of alternans (beats)	(d*V*/d*t*)_max_	872 ± 42	659 ± 41	*p* < 0.0001
	AP latency	928 ± 49	672 ± 35;	*p* < 0.001
	APD_90_	1089 ± 40	868 ± 46	*p* < 0.001
	RMP	852 ± 42	694 ± 44	*p* = 0.01
Maximum duration of alternans episodes (beats)	(d*V*/d*t*)_max_	535 ± 41	404 ± 36	*p* < 0.05
	AP latency	611 ± 46	456 ± 34	*p* < 0.01
	APD_90_	682 ± 57	509 ± 44	*p* < 0.05
	RMP	417 ± 35	312 ± 32	*p* < 0.05

Secondly, in addition to the above effects of genotype alone, genotype and age exerted interacting effects on the incidence of AP latency alternans (*p* < 0.05) and APD_90_ alternans (*p* < 0.05). Young WT hearts had the lowest incidences of AP latency alternans (575 ± 51 beats) with progressive increases in this incidence through the series: young *Pgc-1β* ^*−/−*^ hearts (785 ± 78 beats), aged WT hearts (790 ± 33 beats) and aged *Pgc-1β* ^*−/−*^ hearts (923 ± 81 beats), with the last group showing the greatest amount of AP latency alternans. Post hoc testing demonstrated significantly more AP latency alternans in young *Pgc-1β* ^*−/−*^ hearts compared to that in young WT hearts (*p* < 0.01) and that in aged *Pgc-1β* ^*−/−*^ hearts compared to that in young WT hearts (*p* < 0.001). A similar pattern emerged in post hoc testing for APD_90_ alternans: all remaining groups showed significantly more alternans than young WT hearts (young WT vs young *Pgc-1β* ^*−/−*^, 726 ± 65 vs 1068 ± 53 beats, *p* < 0.001; young WT vs aged WT, 726 ± 65 vs 1040 ± 37 beats, *p* = 0.001; young WT vs aged *Pgc-1β* ^*−/−*^, 726 ± 65 vs 1107 ± 60 beats *p* < 0.0001).

### Alternans at longer BCLs, with longer episodes, and involving multiple AP parameters in *Pgc-1β*^−/−^ hearts

Figure 4 also displays the distribution of alternans in each parameter across different BCLs for each experimental group. It is apparent that there are greater incidences of alternans at longer BCLs in *Pgc-1β* ^*−/−*^ hearts than those in WT hearts. This suggests that they have a greater tendency to instability even at lower heart rates. In addition to the absolute burden of alternans described previously, we quantified the number of discrete sequences of alternans per protocol, the maximum duration of alternans as well as the amount of simultaneous alternans between multiple parameters.

First, there were no distinguishable differences in the number of discrete episodes of alternans in (d*V*/d*t*)_max_, APD_90_ or RMP between young and aged, *Pgc-1β* ^*−/−*^ and WT hearts. In contrast, aged *Pgc-1β* ^*−/−*^ hearts showed a higher number of discrete runs of AP latency alternans than young WT hearts (11.0 ± 1.24 vs 7.43 ± 0.58 runs of alternans; *p* < 0.05).

Secondly, the maximum duration of individual alternans episodes, whether involving (d*V*/d*t*)_max_, AP latency, APD_90_ or RMP, was significantly longer in *Pgc-1β* ^*−/−*^ hearts than that in WT hearts. The maximum duration of alternans episodes involving AP latency also showed significant interactive effects of age and genotype (*p* < 0.05). Post hoc testing demonstrated that young *Pgc-1β* ^*−/−*^ hearts showed longer durations of AP latency alternans than young WT hearts (683 ± 62 vs 406 ± 50 beats; *p* < 0.01).

Thirdly, alternans simultaneously involving different AP characteristics were observed within the same heart. This could involve situations in which the alternating high/low AP latencies or reduced/increased (d*V*/d*t*)_max_ coincided with higher/lower APD_90_ values (Fig. 3c), or in the more pro-arrhythmic pattern, occurred out of phase with higher/lower APD_90_ values (Fig. 3d). *Pgc-1β* ^*−/−*^ hearts showed more frequent simultaneous (d*V*/d*t*)_max_ and APD_90_ alternans compared to WT hearts (72.15 ± 2.22 vs 64.97 ± 3.03% of beats, respectively; *p* = 0.05), with similar occurrences of both patterns of alternans. *Pgc-1β* ^*−/−*^ hearts also showed more frequent simultaneous APD_90_ and AP latency alternans (74.03 ± 2.92 vs 65.11 ± 2.61% of beats; *p* < 0.05). In this case, a greater number of beats assumed the more pro-arrhythmic pattern (697 ± 58 vs 537 ± 43 beats; *p* < 0.05). The incidence of the more pro-arrhythmic pattern of alternans was also significantly affected by interactions between age and genotype (*p* < 0.05). Post hoc Tukey tests demonstrated that young *Pgc-1β* ^*−/−*^ hearts showed higher incidences of simultaneous alternans than young WT hearts (*p* = 0.01).

Finally, we quantified the amplitude of alternans where this occurred, by calculating the mean differences between successive peak and trough values in each parameter. These findings contrastingly demonstrated no significant variations between the groups.

### Altered action potential characteristics in young and aged *Pgc-1β*^−/−^ hearts

**Fig. 5:**
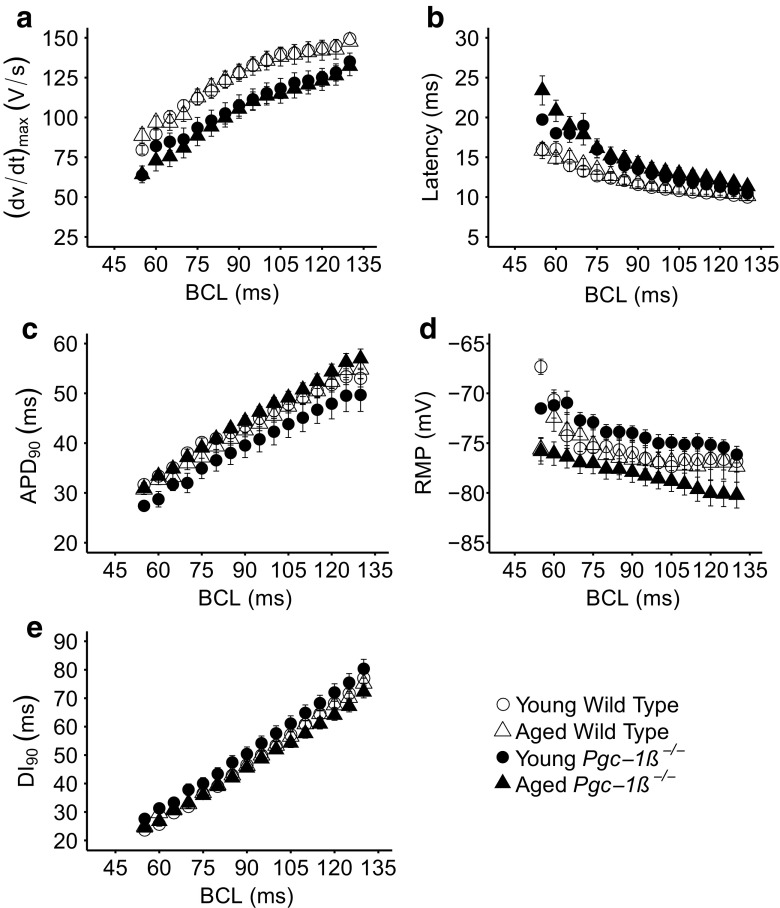
Dependence of maximum rate of AP depolarization, (d*V*/d*t*)_max_ (**a**), AP latency (**b**), APD_90_ (**c**), RMP (**d**) and DI (**e**) on BCL in young and aged, WT and *Pgc-1β* hearts

These differences in electrophysiological stability and pro-arrhythmic tendency were then correlated with differences in AP activation and recovery characteristics over the range of BCLs explored. Figure 5 plots the activation variables of (d*V*/d*t*)_max_ (a) and AP latency (b) as well as the recovery variables of APD_90_, (c) RMP (d) and diastolic interval, DI, (given by the time between the action potential peak and the preceding APD_90_) (e) against BCL in young and aged, *Pgc-1β* ^*−/−*^ and WT hearts. All data varied monotonically with BCL and so their overall magnitudes could be compared by the areas beneath their curves. Table 3 demonstrates that hearts with the *Pgc-1β* ^*−/−*^ genotype have lower (d*V*/d*t*)_max_ values and increased AP latencies relative to WT (both *p* < 0.001). However, there were no detectable effects of either ageing or any interaction between genotype and ageing on (d*V*/d*t*)_max_. In contrast, neither APD_90_ nor RMPs were influenced by genotype alone. However, APD_90_ was longer in aged *Pgc-1β* ^*−/−*^ hearts than that in young *Pgc-1β* ^*−/−*^ hearts (*p* < 0.05), and RMP greater in aged than young hearts.

**Table 3:** Areas under the curves (AUC) of AP parameter with respect to BCL

Parameter	All WT	Young WT	Aged WT	*All Pgc-1β* ^*−/−*^	Young *Pgc-1β* ^*−/−*^	Aged *Pgc-1β* ^*−/−*^	All young	All aged
(d*V*d*t*)_max_ (mV)	8719.17 ± 241.3***	8644.38 ± 315.86	8809.71 ± 380.44	7202.22 ± 323.25***	7118.87 ± 429.06	7272.23 ± 480.81	7916.3 ± 284.69	7936.14 ± 336.1
APD_90_ (ms^2^)	3025.52 ± 77.85	3049.12 ± 111.58	2996.97 ± 109.6	2938.67 ± 101.34	2658.59 ± 153.61†	3173.94 ± 117.74†	2862.73 ± 97.22	3097.52 ± 82.14
DI_90_ (ms^2^)	3425.62 ± 86.84	3432.78 ± 85.07	3416.96 ± 165.08	3470.24 ± 110.69	3608.28 ± 174.96	3354.29 ± 140.05	3516.54 ± 94.39	3381.35 ± 105.68
AP latency (ms^2^)	815.11 ± 29.12***	797.85 ± 38.96	836.02 ± 44.48	994.64 ± 54.38***	970.68 ± 80.8	1014.77 ± 74.79	880.33 ± 45.04	937.58 ± 48.1
RMP (mV)	−5214.08 ± 114.15	−5148.33 ± 149.22	−5293.68 ± 178.95	−5144.06 ± 133.21	−4768.19 ± 91.03	−5459.8 ± 215.23	−4966.9 ± 92.91‡	−5388.06 ± 143.66‡

Table represents the overall magnitude of each parameter across the different experimental categories. Values were obtained by calculating the area under the curves for each parameter in each heart and then means and standard errors calculated. Results of statistical tests are shown. Each symbol represents statistical results from ANOVA; *** denote *p* < 0.001 for independent effects of genotype; ‡ denote *p* < 0.05 for independent effects of age; † denote *p* < 0.05 for interacting effects of genotype and age

### Wavelength as the basis for arrhythmic substrate

The findings above demonstrate pro-arrhythmic features in aged *Pgc-1β* ^*−/−*^ hearts. Further quantification demonstrated increased alternans in both young and aged *Pgc-1β* ^*−/−*^ hearts. This in turn correlated with altered AP activation characteristics as reflected in (d*V*/d*t*)_max_ and AP latency, as opposed to the recovery characteristics, APD_90_ and RMP. The following analyses further tested the hypothesis that activation and not recovery parameters are the primary determinant of arrhythmia in this system.

First, hearts were stratified by arrhythmic and non-arrhythmic phenotypes, regardless of genotype or age. AP parameters observed prior to the onset of either arrhythmia or entry into 2:1 block were then compared. Arrhythmic hearts (*n* = 20) showed lower (d*V*/d*t*)_max_ and longer AP latencies than non-arrhythmic hearts (*n* = 68) ((d*V*/d*t*)_max_, 67.29 ± 6.85 vs 87.66 ± 3.28 V s^−1^, *p* < 0.05; AP latencies 22.25 ± 1.50 vs 18.64 ± 0.88 ms, *p* < 0.05, respectively). Diastolic interval (DI_90_) durations were calculated from the APD_90_ time to the next action potential peak; thus, they were consequently altered as expected from the altered AP rise times (27.43 ± 1.53 vs 31.54 ± 1.09 ms; *p* < 0.05). There were no significant differences between their APD_90_ (32.75 ± 1.22 and 34.79 ± 0.92 ms) and RMPs (−73.81 ± 1.16 vs −73.60 ± 0.72 mV). These findings implicate AP activation rather than recovery parameters in the initiation of arrhythmia.

**Fig. 6:**
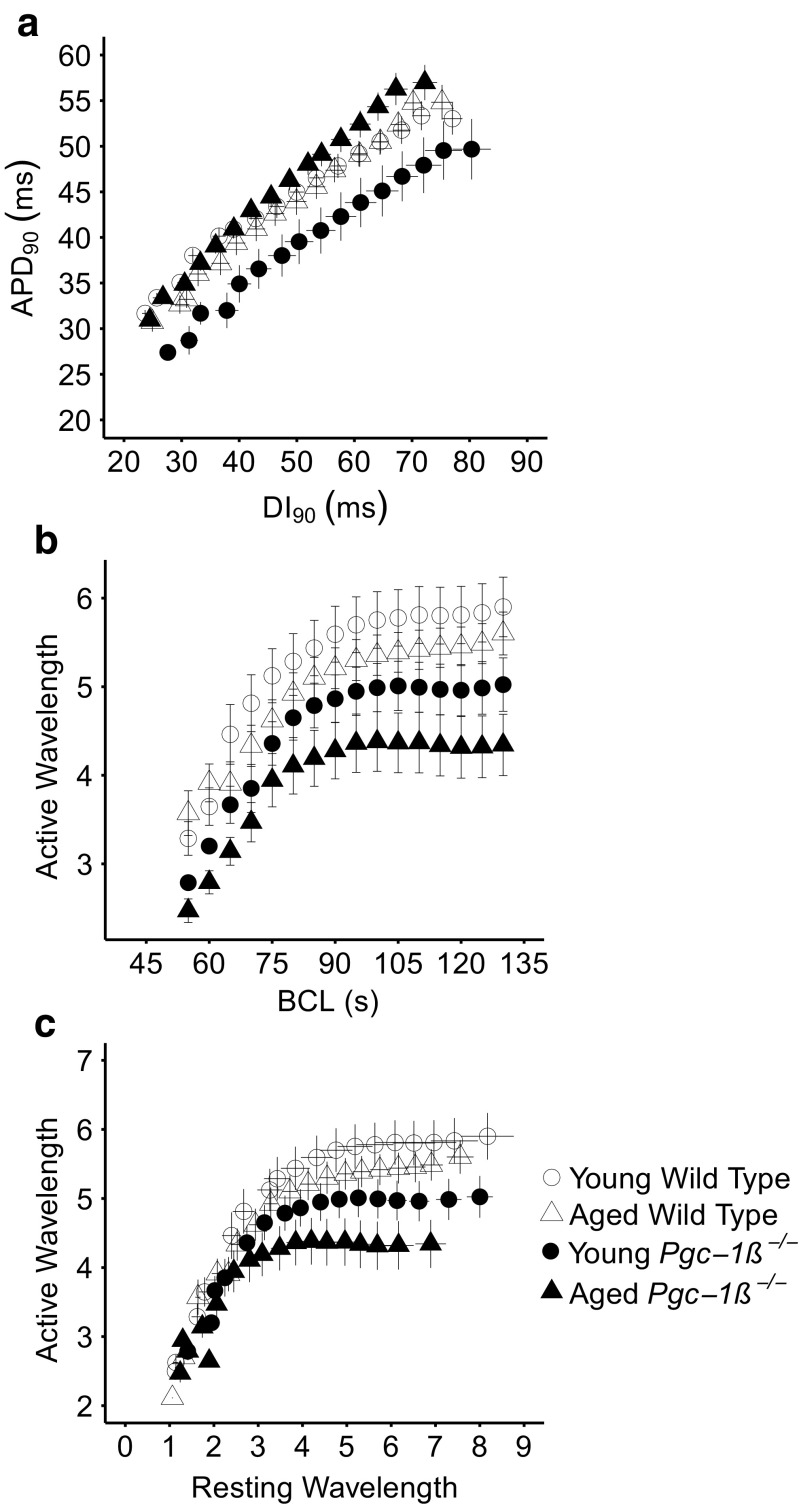
Restitution plots of APD_90_ against DI_90_ (**a**) and of active AP wavelength (**b**) and passive wavelengths (**c**) observed at different BCLs through the incremental pacing procedure in young and aged WT and *Pgc*-*1β* ^*−/−*^ hearts

Secondly, previous reports have correlated alternans characteristics to arrhythmic tendency in both monogenic murine models as well as in clinical situations. Arrhythmic syndromes primarily attributed to repolarisation abnormalities, exemplified by the murine *Scn5a+/∆KPQ* model, were associated with alterations in the relationship between APD_90_ and VERP with varying DI [33, 35, 41]. In contrast, arrhythmia attributed to altered conduction in *Scn5a* ^*+/−*^ hearts was associated with altered relationships between active AP wavelengths, *λ*, and BCL [39] or resting wavelength *λ*_0_ [34]. Figure 6 illustrates the outcome of analyses testing both assumptions. These demonstrated indistinguishable limiting slopes in restitution plots of APD_90_ against DI_90_ in all the four experimental groups (Fig. 6a), in opposition to the prediction from the first hypothesis. We then proceeded to determine *λ*, calculated as the product of conduction velocity represented by 1 / (AP latency) and the corresponding VERP at each BCL. This was derived by interpolating values from the VERP obtained during the S1S2 protocol at a BCL of 125 ms and from the VERP obtained from the BCL during incremental pacing prior to loss of 1:1 stimulus capture. Young and aged WT hearts showed similar *λ*-BCL and *λ*-*λ*_0_ plots (Fig. 6b, c). However, *Pgc-1β* ^*−/−*^ hearts showed lower plateau *λ* values compared to WT hearts, particularly with ageing. This would be consistent with the relative arrhythmic tendencies in the four experimental groups reported here. Further strengthening this hypothesis, the mean wavelengths immediately prior to termination of the protocol into either arrhythmia or 2:1 block were significantly different (*p* = 0.01) at 4.4 ± 0.31 (*n* = 20) for arrhythmic versus 5.4 ± 0.20 (*n* = 68) for non-arrhythmic hearts.

## Discussion

The present study explored pro-arrhythmic phenotypes and their age-dependence in murine hearts deficient in the key mitochondrial upregulator, *Pgc-1β*, previously associated with metabolic dysfunction. Previous molecular and cellular studies in murine *Pgc-1β* ^*−/−*^ cardiomyocytes had confirmed down-regulation of genes related to oxidative phosphorylation, electron transport and the Krebs cycle and upregulation of genes related to cardiac hypertrophy, hypoxia and heart failure [17]. Their fluometric studies demonstrated cellular diastolic Ca^2+^ transients, some forming Ca^2+^ waves producing temporal and spatial Ca^2+^ heterogeneities. Conventional patch clamp studies demonstrated positive voltage shifts in Ca^2+^ current inactivation, increased Na^+^, and transient and inwardly rectifying K^+^ currents under conditions of intracellular Ca^2+^ buffering. These findings together accompanied potentially pro-arrhythmic oscillatory resting potentials, early and delayed afterdepolarisations, and burst action potential firing on sustained current injection potentially related to AP alternans, and ventricular tachycardia [17]. These findings corroborated specifically for the *Pgc-1β* ^*−/−*^ model broader associations between energetic dysfunction and increased reactive oxygen species production [16], the latter affecting voltage-dependent Na^+^ and K^+^ current [32, 48], sarcolemmal K_ATP_ channel function, Na^+^ and Ca^2+^ channel inactivation, late Na^+^ current and ryanodine receptor function [19]. Finally, the accompanying ATP/ADP depletion is known to open sarcolemmal ATP-sensitive K^+^ channels (sarcK_ATP_) [2].

Such changes potentially affect cell-cell coupling [43], AP conduction [32], repolarisation and refractoriness [48], and predispose to alternans and Ca^2+^-mediated pro-arrhythmic triggering [45]. The present experiments now explore and quantify the latter electrophysiological, potentially pro-arrhythmic, consequences in isolated perfused intact murine hearts hitherto employed to explore murine models modelling specific human monogenic channelopathies. [19]. Utilisation of intracellular cardiomyocyte recordings in this study made it possible to determine resting membrane potentials (RMPs), AP amplitudes, and provided sufficient bandwidth to follow maximum rates of AP depolarisation (d*V*/d*t*)_max_, measurements not possible with monophasic action potential recordings used previously. Additional simultaneous whole-heart ECG recordings allowed correlation of these cellular findings with arrhythmic events at the whole-organ level. Finally, use of additional experimental groups made it possible to assess and compare effects of ageing on the *Pgc-1β* ^*−/−*^ and WT phenotypes.

This study is the first to establish an age-dependent pro-arrhythmic phenotype in *Pgc-1β* ^*−/−*^ murine hearts and the electrophysiological basis for this. The pro-arrhythmic phenotype shown by aged *Pgc-1β* ^*−/−*^ hearts parallels epidemiological clinical data reporting age-dependent arrhythmic risks in metabolic disease. Early or delayed afterdepolarisation phenomena, previously implicated in arrhythmic triggering processes [6, 46], were not observed, making a dependence of the arrhythmic phenotype on such triggering events less likely.

*Pgc-1β* ^*−/−*^ hearts irrespective of age showed an increased incidence of alternans in AP activation ((d*V*/d*t*)_max_ and AP latency) and recovery (APD_90_ and RMP) parameters. These occurred at lower pacing frequencies than in WT hearts, consistent with greater instability in hearts with metabolic compromise. Arrhythmic substrate resulting from repolarisation abnormalities gives restitution plots of APD_90_ against DI_90_ with distinct gradient properties from those of WT hearts. However, the restitution loci in *Pgc-1β* ^*−/−*^ hearts showed limiting slopes indistinguishable from WT hearts. Instead, *Pgc-1β* ^*−/−*^ hearts showed compromised AP activation as reflected in (d*V*/d*t*)_max_ and AP latency, and shorter effective refractory periods than WT hearts. Furthermore, the onset of arrhythmia was associated with compromised AP activation but normal AP recovery.

These findings localise the arrhythmic substrate to defects in AP activation. *Pgc-1β* ^*−/−*^ hearts had altered AP wavelength (*λ*) properties, determined from the conduction velocity—effective refractory period product. Young and aged WT hearts showed similar *λ*-BCL and *λ*-*λ*_0_ plots whereas young and aged *Pgc-1β* ^*−/−*^ hearts showed reduced values of *λ* through all BCLs—an effect more marked in the aged *Pgc-1β* ^*−/−*^ hearts. This finding is an exact parallel of previous demonstrations of the presence of arrhythmic phenotypes and of pro-arrhythmic alternans phenomena.

The present observations in *Pgc-1β* ^*−/−*^ hearts parallel features reported in *Scn5a* ^*+/−*^ and *RyR2*-P2328S/P2328S cardiac models for Brugada syndrome and catecholaminergic polymorphic ventricular tachycardia, respectively [33–35, 39]. Recent work had similarly attributed arrhythmic substrate in *RyR2*-P2328S/P2328S hearts to slowed AP conduction accompanying reductions in (d*V*/d*t*)_max_ [53] and in Na^+^ currents to extents comparable to those observed in Na_V_1.5-haploinsufficient *Scn5a* ^+/−^ hearts [26, 27]. These reductions in Na^+^ currents in the *RyR2*-P2328S/P2328S cardiomyocytes were attributed to effects of altered Ca^2+^ homeostasis upon Na_v_1.5 expression [39] and/or acutely upon Na_v_1.5 function [26, 27]. *RyR2*-P2328S/P2328S and *Pgc-1β* ^*−/−*^ cardiomyocytes share abnormal Ca^2+^ handling phenotypes [17]. The normal or even enhanced Na^+^ currents in patch-clamped *Pgc-1β* ^*−/−*^ cardiomyocytes [17] are compatible with acute effects of their altered Ca^2+^ homeostasis upon membrane excitability in cardiomyocytes in intact tissue [26, 31, 39, 42]. Thus, patch-clamped WT myocytes also show respective acutely reduced, or increased, Na^+^ current and (d*V*/d*t*)_max_, with increases in, or sequestration of, the pipette [Ca^2+^] [7]. These effects could reflect Ca^2+^-Na_v_1.5 interactions involving direct Ca^2+^ Na_v_1.5 binding at an EF hand motif close to the Na_v_1.5 carboxy-terminal [50] or indirect Ca^2+^ binding involving an additional ‘IQ’ domain binding site for Ca^2+^/CaM in the Na_v_1.5 C-terminal region [15, 37, 47]. The present findings thus throw light on possible, more widespread, effects of altered intracellular Ca^2+^ homeostasis on AP propagation in addition to identifying electrophysiological mechanisms underlying the arrhythmic risk associated with metabolic disturbance.
